# Down-Regulation of KCC2 Expression and Phosphorylation in Motoneurons, and Increases the Number of in Primary Afferent Projections to Motoneurons in Mice with Post-Stroke Spasticity

**DOI:** 10.1371/journal.pone.0114328

**Published:** 2014-12-29

**Authors:** Takuya Toda, Kazuto Ishida, Hiroshi Kiyama, Toshihide Yamashita, Sachiko Lee

**Affiliations:** 1 Department of Physical and Occupational Therapy, Graduate School of Medicine, Nagoya University, Nagoya, Japan; 2 Department of Functional Anatomy and Neuroscience, Graduate School of Medicine, Nagoya University, Nagoya, Japan; 3 Department of Molecular Neuroscience, Graduate School of Medicine, Osaka University, Osaka, Japan; Inserm, France

## Abstract

Spasticity obstructs motor function recovery post-stroke, and has been reported to occur in spinal cord injury and electrophysiological studies. The purpose of the present study was to assess spinal cord circuit spasticity in post-stroke mice. At 3, 7, 21, and 42 d after photothrombotic ischemic cortical injury in C57BL/6J mice, we observed decreased rate-dependent depression (RDD) of the Hoffmann reflex (H reflex) in the affected forelimb of mice compared with the limbs of sham mice and the non-affected forelimb. This finding suggests a hyper-excitable stretch reflex in the affected forelimb. We then performed immunohistochemical and western blot analyses to examine the expression of the potassium-chloride cotransporter 2 (KCC2) and phosphorylation of the KCC2 serine residue, 940 (S940), since this is the main chloride extruder that affects neuronal excitability. We also performed immunohistochemical analyses on the number of vesicular glutamate transporter 1 (vGluT1)-positive boutons to count the number of Ia afferent fibers that connect to motoneurons. Western bolts revealed that, compared with sham mice, experimental mice had significantly reduced KCC2 expression at 7 d post-stroke, and dephosphorylated S940 at 3 and 7 d post-stroke in motoneuron plasma membranes. We also observed a lower density of KCC2-positive areas in the plasma membrane of motoneurons at 3 and 7 d post-stroke. However, western blot and immunohistochemical analyses revealed that there were no differences between groups 21 and 42 d post-stroke, respectively. In addition, at 7 and 42 d post-stroke, experimental mice exhibited a significant increase in vGluT1 boutons compared with sham mice. Our findings suggest that both the down-regulation of KCC2 and increases in Ia afferent fibers are involved in post-stroke spasticity.

## Introduction

Spasticity, a common disorder in patients with brain and spinal cord injuries, is characterized by a velocity-dependent increase in muscle tone resulting from a hyper-excitable stretch reflex [1]–[2]. An estimated 42.6% of patients with stroke exhibit spasticity 6 months post-stroke, and this disrupts their ability to perform activities of daily living and decreases their quality of life [3]–[5].

Studies of spasticity have assessed human patients, as well as animal models of spinal cord injury and stroke. These previous studies reported that the underlying mechanisms of spasticity include increased motoneuron excitability [6]–[7], increased excitatory synaptic inputs associated with Ia afferent fibers [8], and decreased inhibition of the spinal network [9]–[11]. Thus, the mechanisms of spasticity caused by stroke and spinal cord injury may be similar. However, the mechanisms of post-stroke spasticity remain unclear due to limitations associated with assessing human patients. Moreover, there is a general lack of spasticity in post-stroke animal models. This latter drawback can now be overcome since we have recently established a novel post-stroke mouse model of spasticity [12].

The potassium-chloride cotransporter 2 (KCC2) is a major chloride extruder that is critical for maintaining chloride ion homeostasis in mature neurons [13]. KCC2 maintains the low intracellular chloride concentration that is necessary for the hyperpolarizing actions of the inhibitory neurotransmitters, “Insert>Symbols” γ -aminobutyric acid (GABA) and glycine, in mature neurons. KCC2 transporter function regulates the expression and phosphorylation of serine, threonine, and tyrosine of KCC2 in the plasma membrane [14]. It is well-established that stability of the cell surface is regulated by the phosphorylation of the serine 940 residue in a protein kinase C-dependent manner [15]. Moreover, dephosphorylation of KCC2 serine 940 has been shown to result in N-Methyl-D-aspartic acid (NMDA) receptor activity and Ca^2+^ influx, leading to enhanced neuronal activity [16]. Recently, it was reported that spinal cord injury induced a down-regulation of KCC2 in motoneurons, leading to spasticity [17]. KCC2 down-regulation has also been reported in other central nervous system disorders, such as seizures [18], neuropathic pain [19], amyotrophic lateral sclerosis [20], and cerebral ischemia [21]. We hypothesized that one of the mechanisms of post-stroke spasticity is that KCC2 expression in affected spinal motoneurons is decreased after stroke, while synaptic inputs associated with Ia afferent fibers are increased.

Here, we describe immunohistochemical and western blot evidence indicating decreased KCC2 expression, serine 940 dephosphorylation in motoneurons, and pathological Ia afferent plasticity in a mouse model of post-stroke spasticity. Our findings suggest that these changes may be involved in the development of post-stroke spasticity.

## Materials and Methods

### Animals

Adult male C57BL/6J 77 mice weighing 25–30 g (SLC, Shizuoka, Japan) were used. Mice were housed in groups of 4–6 animals per cage under a 12-h light dark cycle. Food and water were supplied ad libitum. All procedures were approved by Nagoya University Animal Experiment Committee (Permit Number: 022–035).

### Photothrombotic stroke model

**Figure 1:**
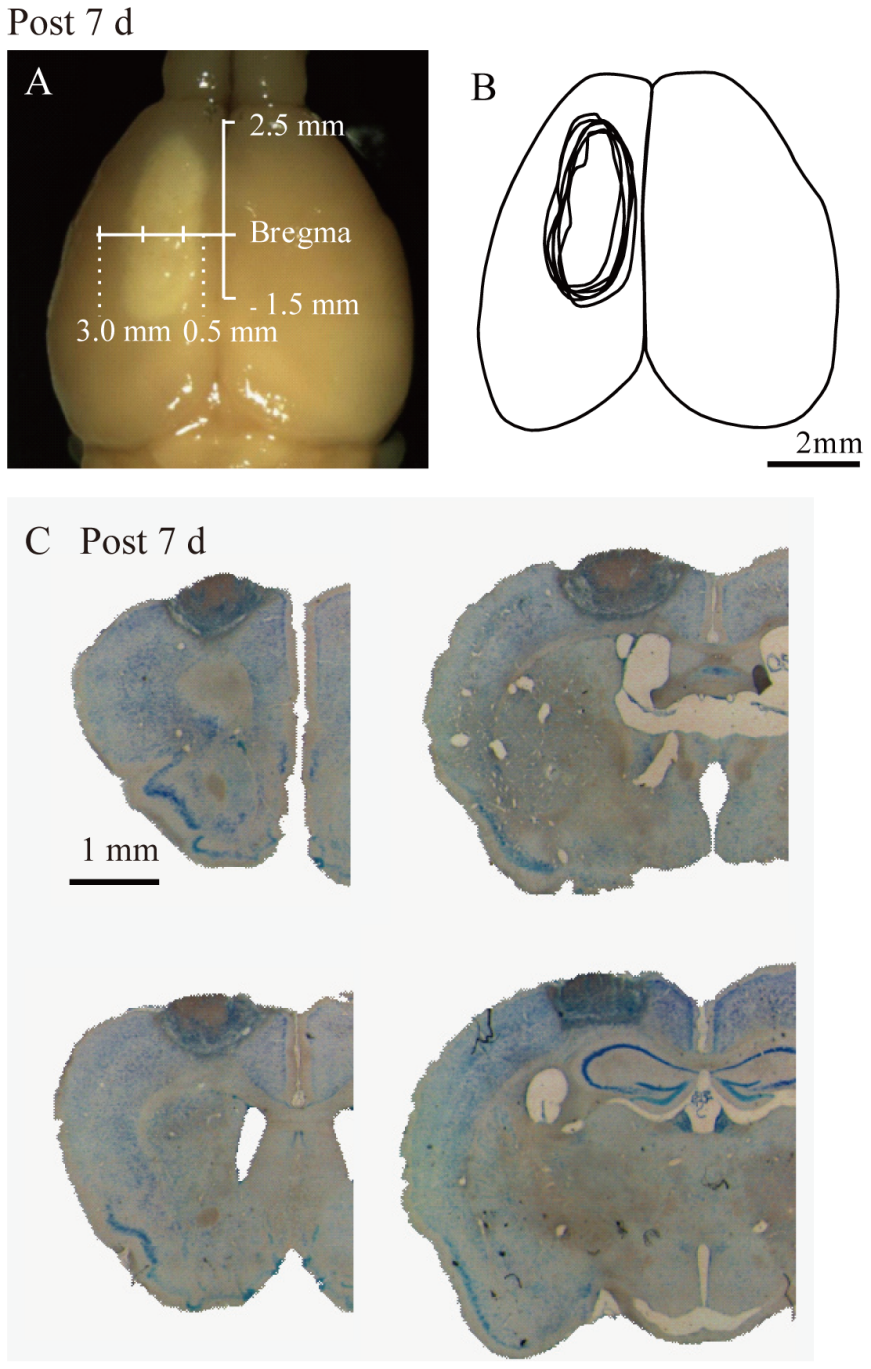
Photothrombotic stroke lesion in the left rostral and caudal forelimb motor cortex. A: Picture of a lesion at 7 d after stroke. B: A tracing of lesion areas (7 d post-stroke, n = 6). C: Nissl staining of coronal sections of the brain at 7 d post-stroke. The left rostral and caudal forelimb motor cortex show specific injuries. The mean injury volume was 5.13±1.23 mm^3^ (n = 6).

Focal cortical ischemia was induced by microvessel photothrombosis, as described previously [12]. Mice (stroke: 39, sham: 38) were anesthetized with intraperitoneal (i.p.) sodium pentobarbital (Somnopentyl, 50 mg/kg body weight; Kyoritsu Seiyaku, Tokyo, Japan) and were placed in a stereotaxic instrument. The skull surface was exposed with a midline incision made on the scalp. Rose Bengal (10 mg/ml solution in 0.9% saline, 0.03 mg/g body weight, Sigma, St. Louis, Mo, USA) was injected into the tail vein and a light from a fiber optic bundle of a cold light source (step 34, 184–204 lm, CL 6000 LED, Zeiss, Oberkochen, Germany) was focused on the skull for 15 min. The light beam was centered 2.5 mm anterior to 1.5 mm posterior and 0.5 to 3.0 mm lateral to the bregma to induce a thrombotic lesion in the left rostral and caudal forelimb motor cortex, where Fulton and Kennard demonstrated brain lesions to induce spasticity (Fig. 1A)[22]–[23]. The scalp was sutured, and animals were allowed to regain consciousness. Animals were random selected and sham animals received the same injection of Rose Bengal, but were not exposed to a light beam.

### Electrophysiological assessment of spasticity

Spasticity was assessed by the H reflex, which was measured using a previously described electrophysiological procedure [12], [17], [24]. Briefly, 21 mice (stroke: 11, sham: 10) were anesthetized with ketamine (200 mg/kg i.p., additional dosage: 1/2 initial dose every 45 min, CS pharmaceutical Co., Aichi, Japan) and their fore- and hindlimbs were fixed to an aluminum plate with plastic tape. The aluminum plate was placed on a warm pad to maintain the animal's body temperature around 37°C. A pair of stainless needle electrodes were transcutaneously inserted to stimulate nerve bundles, including the ulnar nerve at the axilla, with a stimulator (1–3 mA in increments of 0.1 mA, SEN-7103, Nihon Kohden Co., Aichi, Japan). The H reflex was recorded at both the abductor digiti minimi muscles with an amplifier (High pass 0.1 kHz, SS-201J, Nihon Kohden Co.) and A/D converter (low pass 1 kHz, PowerLab, Dunedin, New Zealand). To determine the minimum intensity required to evoke the H reflex, nerves were electrically stimulated (duration: 0.2 ms) at 0.1 Hz with increasing current intensities. The nerves were then stimulated 23 times each at 0.1, 0.5, 1, 2, and 5 Hz to measure the RDD of the H reflex. To avoid the effect of repeated tests, 2-min resting intervals were provided between each frequency. To compare RDDs of the H reflex at different frequencies, we calculated the means of the differences between the maximum and minimum of the H reflex amplitudes with PowerLab systems and Scope software (version 4.0, AD Instruments).

### Immunohistochemistry

For immunohistochemical assays, mice were deeply anesthetized with intraperitoneal sodium pentobarbital and perfused with 50 ml of 4% paraformaldehyde (PFA) solution in 0.1 M phosphate buffer (PB) at 3, 7, and 42 d after stroke. The number of animals per time period were as follows: stroke and sham animals: post 3 d n = 3, post 7 d n = 4, post 42 d n = 3. For KCC2 labeling, 50 motoneurons on affected and non-affected sides were measured for each sham and stroke animal. Similarly, the number of animals and motoneurons assessed in vGluT1 labeling for sham and stroke groups was n = 3 per time-point (3, 7, and 42 d post-stroke) and 50 motoneurons on the affected and non-affected sides. Spinal cords (C4–C7) and brains were removed and post-fixed in 4% PFA overnight at 4°C. Samples were stored at −80°C following immersion in 30% sucrose for 3 d at 4°C for cryoprotection. Frozen samples embedded in optimal cutting temperature compound (Tissue-Tek, Sakura Finetek Japan, Tokyo, Japan) were cut into 20 “Insert>Symbols” µm-thick sections.

Sections were blocked with blocking buffer (5% normal donkey or goat serum/0.1% triton X-100/phosphate-buffered saline [PBS]) for 1 h following incubation in 0.1% trypsin/PBS for 5 min at room temperature. We incubated sections with goat anti-ChAT (1∶200, Millipore, Cat# AB144P, RRID:AB_262156), rabbit anti KCC2 (1∶500, Millipore, Cat# 07-432, RRID:AB_310611), and guinea pig anti-vGluT1 (1∶500, Synaptic Systems, Cat# 135304, RRID: AB_887878) as primary antibodies overnight at 4°C. The sections were then washed three times with 0.05% Tween 20/PBS (PBST), followed by incubation with secondary antibodies for 1 h at room temperature. Alexa 488 or 568 conjugated with of donkey anti-goat, goat anti-rabbit, and goat anti-guinea pig immunoglobulins (Invitrogen, 1∶250 for donkey anti-goat, RRID: AB_10564097, 1∶500 for goat anti-rabbit and goat anti-guinea pig, RRID:AB_10893781 and AB_10894727, respectively) were used as secondary antibodies. Sections were then counterstained with 4′, 6-diamidino-2-phenylindole (DAPI; 1 “Insert>Symbols” µg/ml; Santa Cruz, Biotechnology, Santa Cruz, USA), and mounted with fluorescence mounting medium (S3023, DAKO, Glostrup, Denmark).

### Histological quantification

To measure KCC2 expression in spinal motoneuron plasma membranes, six stained transverse sections (C4 to C7) were photographed under a confocal laser-scanning microscope (Z stack image of step 0.15 “Insert>Symbols” µ m, ×400, A1, Nikon, Tokyo, Japan) and KCC2 locations within membrane and cytosol regions were confirmed with XYZ cross sections.

KCC2-positive areas were measured at the plasma membranes of 50 motoneurons on the affected and non-affected sides of each animal using adjusted threshold control in ImageJ software for elimination of background (version 1.47v, National Institute of Health, Bethesda, MD, USA). To determine differences in KCC2-positive areas, KCC2-labeled pixels were divided with motoneuron somatic perimeters, which enabled us to then calculate the ratios of pixels per perimeter [21], [25].

For measurements of vGluT1-positive afferent fibers on motoneurons, the six stained sections from each level of C4 to C7 were photographed with a Z-stack tool (0.7 “Insert>Symbols” µ m/step, 15 steps/section) under confocal laser-scanning microscopy, and vGluT1-positive boutons were counted in 50 motoneurons of the affected and non-affected sides per animal [8], [26]. The number of vGluT1 boutons in contact with ChAT-positive motoneurons was counted using Z-stack images.

### Western blot analysis

The amount of KCC2 and phosphorylated serine 940 (S940) of KCC2 in the cervical spinal cord was determined by western blot, as described previously [27]. At 3, 7, and 21 d post-stroke, mice (n = 36) were deeply anesthetized and transcardially perfused with chilled saline. Spinal cords (C4 to C7) were rapidly removed, and ventral columns in the affected and non-affected sides were sampled. Samples were processed in cold homogenization buffer (320 mM sucrose in Tris-HCl [pH 7.5], and a mixture of protease and phosphatase inhibitors) and centrifuged at 7,000× g for 5 min at 4°C. Supernatants were again centrifuged at 18,000× g for 70 min at 4°C [21], [27], and precipitations were collected as membrane-enriched fractions. The collected pellets were dissolved in lysis buffer (1% IGEPAL CA-630, 0.1% sodium dodecyl sulfate [SDS], and a mixture of protease and phosphatase inhibitors), and supernatants were collected as cytoplasmic fractions. We then determined protein concentrations using a bicinchoninic assay (Thermo Fisher, Waltham, MA, USA). Samples were separated in 10% SDS-polyacrylamide gel, and proteins were transferred to polyvinylidene fluoride membranes. The membranes were incubated for 1 h in 5% non-fat dry milk/PBST at room temperature and then incubated overnight at 4°C with the following primary antibodies: rabbit anti-KCC2 (1∶1000), rabbit anti-Phospho-Ser^940^ KCC2 (1∶1000, Phosphosolutions, Cat# p1551-940), mouse anti- tubulin (1∶1000, Santa Cruz Biotechnology Cat# sc-5286), and goat anti- NKCC1 (1∶200, Santa Cruz Biotechnology, Cat# sc-21545, RRID: AB_2188633). For secondary antibodies, horseradish peroxidase-conjugated, anti-rabbit immunoglobulin (1∶5000, Cell Signaling Technology, Cat# 7074, RRID: AB_2099233) and anti-mouse immunoglobulins (1∶1000, Cell Signaling technology, Cat# 7076, RRID: AB_330924), and anti-goat immunoglobulins (1∶1000, Santa Cruz Biotechnology, Cat# sc-2020, RRID: AB_631728) were used, and the membranes were incubated for 1 h at room temperature. After each step, membranes were washed with PBST three times for 10 min each. Proteins were detected using an enhanced chemiluminescence method (Luminata Forte Western HRP Substrate, Merck Millipore). The optical densities of the protein bands were quantified using ImageJ software (National Institute of Health).

### Statistical analyses

Data were reported as group mean values ± standard error of means (S.E.M.). All data were confirmed and equality of variance was found using F-statistics. Statistical significance was determined using a one-way analysis of variance (ANOVA) followed by Turkey-Kramer's post hoc tests. P<0.05 was considered significant.

## Results

### Cortical infarct lesion location and volume

We induced focal ischemia by microvessel photothrombosis in the motor area of the cerebral cortex of mice. The average lesion volume, as assessed by Nissl-stained sections at 7 d post-stroke, was 5.13±1.23 mm^3^ (n = 6). Lesions in the rostral and caudal forelimb motor areas were reproducible (Fig. 1A-C).

### Electrophysiological spasticity assessment

**Figure 2:**
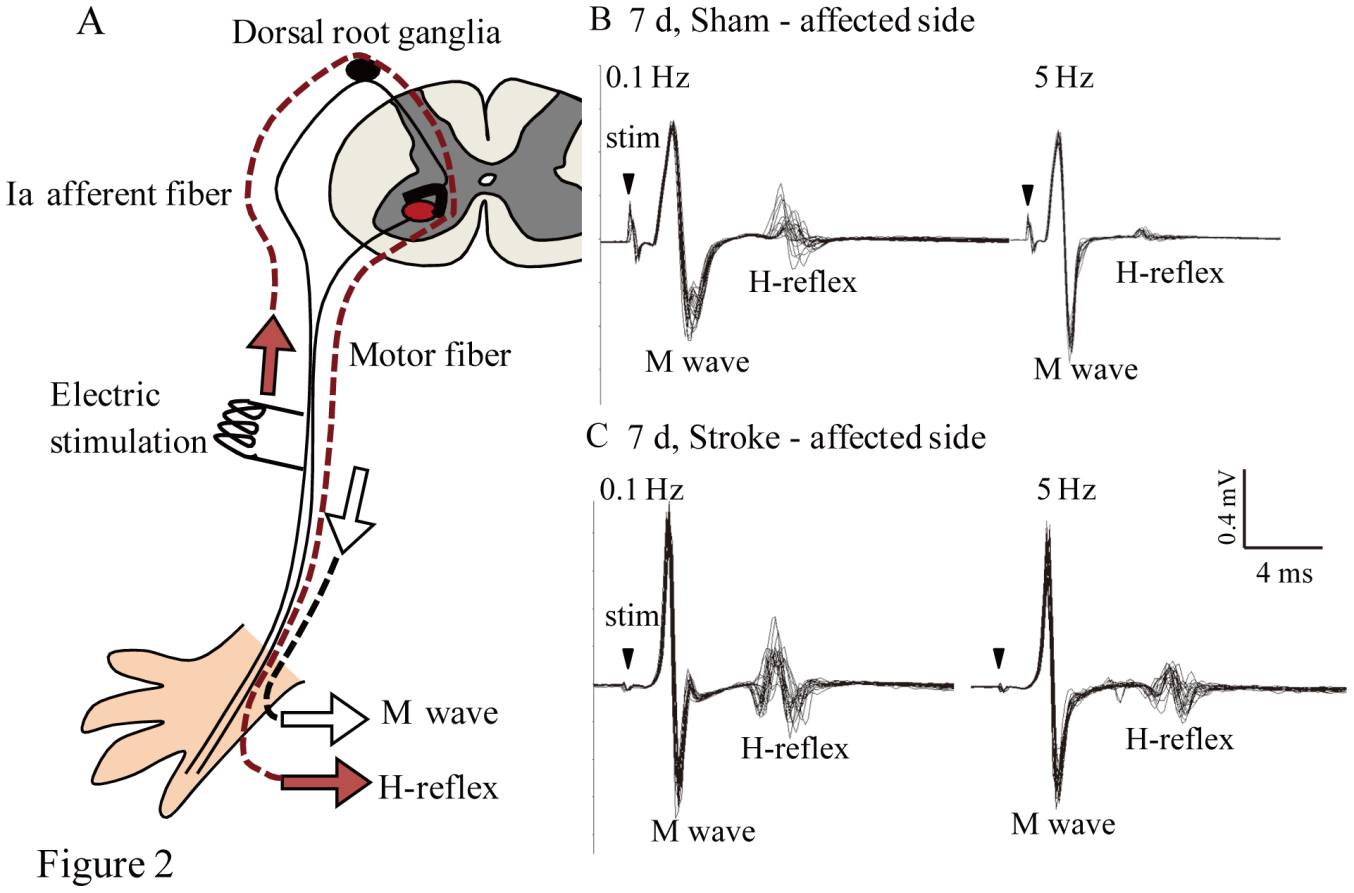
The RDD of the H reflex. A: An illustration of the H reflex in the spinal cord circuit. The M wave is produced by orthodromically transmitting stimulation to motor fibers. The H reflex is a monosynaptic reflex that is transmitted via Ia afferent fibers. B and C: Evoked electromyograms measured in sham mice (B) and on the affected forelimb of stroke mice (C) at 7 d post-stroke. Repeated stimulation at high frequency (5 Hz) decreased the amplitude of the H reflex in sham mice (B). The amplitude of the H reflex on the affected forelimb of stroke mice was not decreased following 5 Hz stimulation (C).

**Figure 3:**
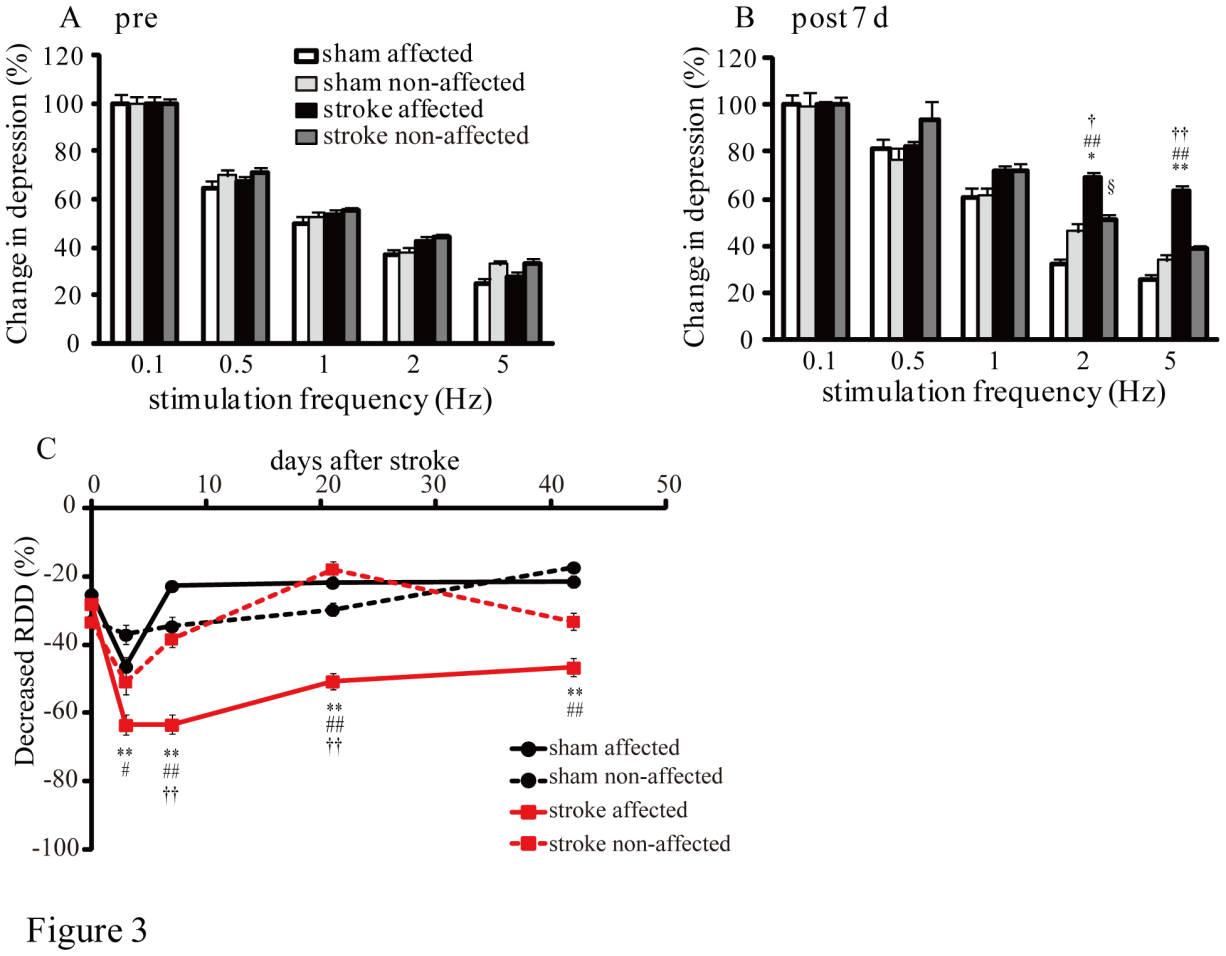
Changes in H reflex RDDs after stroke. A: RDDs of the H reflexes at baseline (pre-stroke). B: Changes in RDDs of the H reflex 7 d post-stroke in sham and stroke animals. Data are presented as percentages relative to the mean amplitude at 0.1 Hz in the same test. C: Changes in RDDs of the H reflex with 5 Hz stimulation on the affected and non-affected sides in stroke and sham mice. Error bars indicate S.E.M. One-way ANOVA with post hoc Tukey-Kramer tests, ##p<0.01, between stroke-affected and sham-affected sides, *p<0.05 and **p<0.01, between stroke-affected and sham non-affected sides, †*p*<0.05 and ††*p*<0.01, between stroke-affected and stroke non-affected, §*p*<0.05, between stroke non-affected and sham-affected sides.

We analyzed the RDD of H reflexes to confirm spasticity development at 3, 7, 21, and 42 d post-stroke (post 3d: sham  = 10, stroke  = 11, post 7 d: sham  = 10, stroke  = 8, post 21 d: sham  = 5, stroke  = 7, post 42 d: sham  = 4, stroke  = 8). We monitored the M wave, which is produced by transmitting orthodromic stimulation to the muscle fiber, and the H reflex by stimulating nerve bundles including the ulnar nerve (Fig. 2A). The H reflex is a monosynaptic reflex that is transmitted via Ia afferent fibers, thus, the latency of the H reflex (6–8 ms) is longer than that of the M wave (2–4 ms) (Fig. 2B, C)[12], [28]. Under normal conditions, we observed a decrease in H reflex amplitude (RDD) with high-frequency (5 Hz) stimulation (Fig. 2B). Notably, the H reflex amplitude was not decreased with 5 Hz stimulation in the affected side after stroke (Fig. 2C). The decrease in RDDs post-stroke confirmed the induction of spasticity since no change in H reflex RDDS was observed in animals under control conditions (Fig. 3A). At 7 d post-stroke, RDDs of the H reflex of the stroke-affected side were significantly decreased at 2 and 5 Hz frequency stimulations compared to the stroke non-affected side and the sham-affected and non-affected side (2 Hz: *p<0.05, vs. sham affected, ##p<0.01, vs. sham non-affected, †p<0.05, p<0.01 in all comparison, Fig. 3B). In addition, the RDDs of the H reflex in the stroke non-affected side were significantly decreased at 2 Hz stimulations compared to the sham-affected side (§p<0.05, Fig. 3B). We also compared H reflex RDDs elicited by 5 Hz of stimulation in the affected and non-affected sides of both sham and stroke mice and found that the RDDs in the stroke-affected side were significantly decreased at all post-stroke time points compared to sham animals (3 d, #<0.05, vs. sham non-affected, p<0.01, in other comparison, Fig. 3C). Moreover, significant decreases of RDDs in the stroke-affected side were found at 7 and 21 d compared to the stroke non-affected side (p<0.01, Fig. 3C).

### Post-stroke down-regulation of KCC2 expression in motoneuron plasma membranes

**Figure 4:**
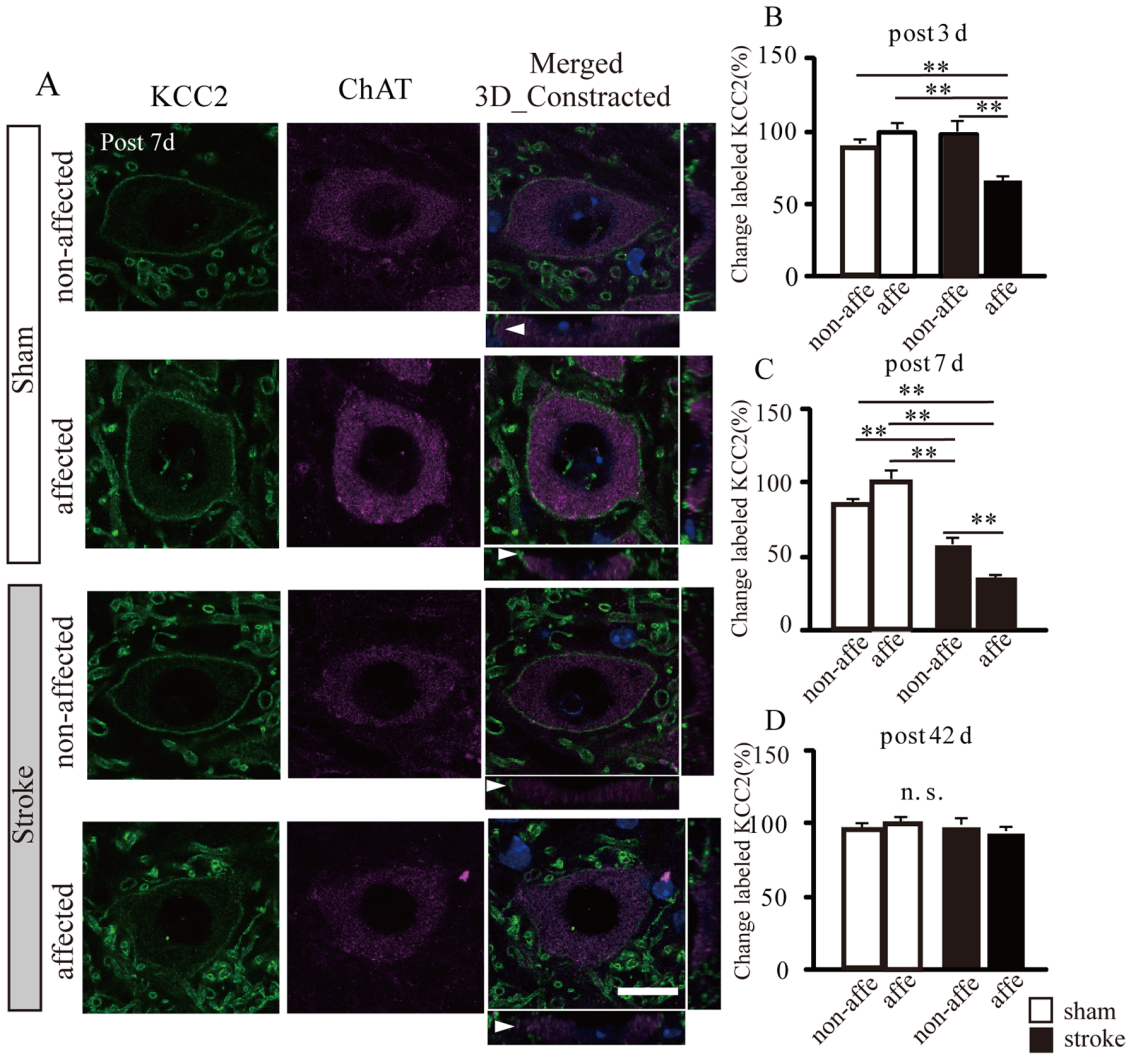
Down-regulation of KCC2 levels using immunohistochemical analysis in motoneuron plasma membrane after stroke. A: Immunofluorescence photomicrographs captured with confocal microscopy showing individual motoneurons labeled with ChAT in magenta, KCC2 in green, and DAPI in blue. Merged image was reconstructed to three dimensional (3D) Z stack images. Arrowheads show the discontinuous labeling of KCC2 in the plasma membrane. Scale bar = 20 “Insert>Symbols” µ m. B-D: Semi-quantification of the density of KCC2 labeling in plasma membranes of spinal motoneurons. Labeled KCC2 is shown as a percentage of the pixel surface per somatic perimeter in the sham-affected side at 3, 7, and 42 d post-stroke, respectively.

We analyzed KCC2 expression in plasma membranes by immunohistochemistry and western blot analyses. Choline acetyltransferase (ChAT) was used as a motoneuron marker for immunohistochemical analyses (Fig. 4A). Although KCC2 proteins are primarily expressed at neuronal plasma membranes and in dendritic spines and shafts [29]–[30], KCC2 in the plasma membrane is responsible for maintaining chloride ion homeostasis in the soma. The function of KCC2 in dendritic shafts is unclear [31]. Therefore, we measured the KCC2-positive area in plasma membranes at 3, 7, and 42 d after stroke (50 motoneurons on affected and non-affected sides/animal).

We found a significant decrease in KCC2-positive areas in plasma membranes of the stroke-affected side at 3 and 7 d compared to both sides in sham animals and the non-affected side of stroke mice (3 d: sham-affected: 100±4.1%, sham non-affected: 89.1±3.4%, stroke-affected: 64.9±3.5%, stroke non-affected: 97.3±7.1%, 7 d: 100±3.7%, sham non-affected: 91.7±4.3%, stroke-affected: 35.5±1.8%, stroke non-affected: 63.5±5.3%, p<0.01 in all comparison, Fig. 4B, C). In the non-affected side of stroke mice at 7 d post-stroke, the KCC2-positive areas was significantly decreased compared to sham mice (p<0.01, Fig. 4C). However, there were no significant differences in KCC2-positive areas between sham and stroke mice at 42 d after stroke (42 d: sham affected: 100±3.5%, sham non-affected: 98.0±3.1%, stroke-affected: 92.1±3.5%, stroke non-affected: 96.4±4.0%, Fig. 4D), but there was a significant decrease in H reflex RDDs in the affected side of stroke mice compared to sham mice (p<0.01, Fig. 3C).

To identify changes in expression levels of KCC2 and phosphorylated S940 in KCC2 in the membrane fraction, we performed western blotting 3, 7, and 21 d post-stroke. We also assessed sodium-potassium-chloride cotransporter (NKCC1), which allows chloride ions to enter cells [32].

**Figure 5:**
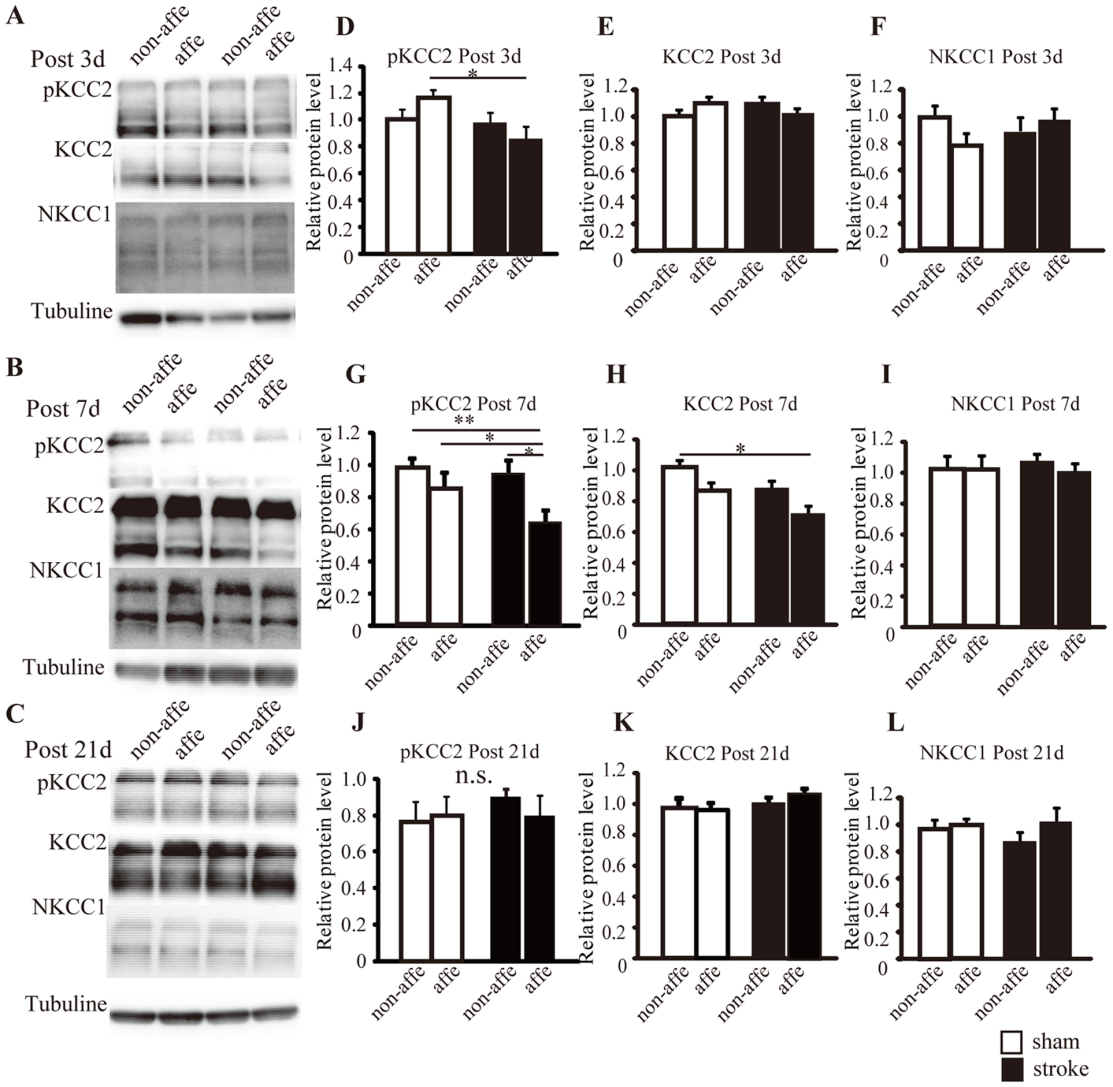
KCC2, S940 phosphorylation in KCC2, and NKCC1expression levels assessed by western blot analysis in membrane-enriched fractions of the ventral horn. A, B, and C: At 3, 7, and 21 d post-stroke, bands of KCC2, phosphorylated S940, NKCC1, and tubulin. D-L: Quantification analysis of KCC2, phosphorylated S940, NKCC1levels at 3, 7, and 21 d after stroke. White bars are sham animals, and black bars are stroke animals. Data are presented as relative ratio to the mean sham non-affected and as the mean ± S.E.M. One-way ANOVA post hoc test Tukey-Kramer tests: *p<0.05, **p<0.01.

Total KCC2 expression levels at the membrane fraction of the stroke affected side was significantly reduced compared to sham animals and observed to decline to72.5±5.1% in the sham non-affected side at 7 d post-stroke (KCC2: sham non-affected: 100.0±0%, sham-affected: 87.1±4.3%, stroke non-affected: 86.9±5.1%, p<0.05, Fig. 5G). At 3 and 21 d post-stroke, there was no significant difference in expression between the stroke-affected side of sham animals and the stroke non-affected side (Fig. 5D and J).

In terms of phosphorylated S940, a significant decline of S940 phosphorylation on the stroke-affected side was detected compared to the sham-affected side at 3 d post-stroke (p<0.05, Fig. 5E). This decline was also observed on both sham sides (to non-affected, p<0.01; to affected, p<0.05; Fig. 5 H) and the stroke non-affected side (p<0.05, Fig. 5H) (S940 pKCC2 at post 3 d: sham non-affected: 100.0±0%, sham-affected: 113.4±5.1%, stroke non-affected:95.3±6.9%, stroke-affected:84.2±8.2, Fig. 5E)(S940 pKCC2 at post 7 d: sham non-affected: 100.0±0%, sham-affected: 87.8±6.4%, stroke non-affected:86.2±6.3%, stroke-affected:63.3±6.1, Fig. 5H). At 21 d post-stroke, there was no significant difference detected in any of the groups (Fig. 5K).

In contrast, NKCC1 expression was not significantly different at 3, 7, and 21 d between sham and stroke mice (Fig. 5F, I, L). We also observed no difference in the expression of tubulin between any of the animals.

### Increase in the number of vGluT1-positive boutons on motoneurons after stroke

Next, we analyzed the number of vesicular glutamate transporter 1(vGluT1)-positive boutons in contact with ChAT-positive motoneurons to determine if there was an increase in the number of primary afferent fiber (e.g., Ia fibers) synapses. This was done by capturing Z-stack images using confocal laser-scanning microscopy.

**Figure 6:**
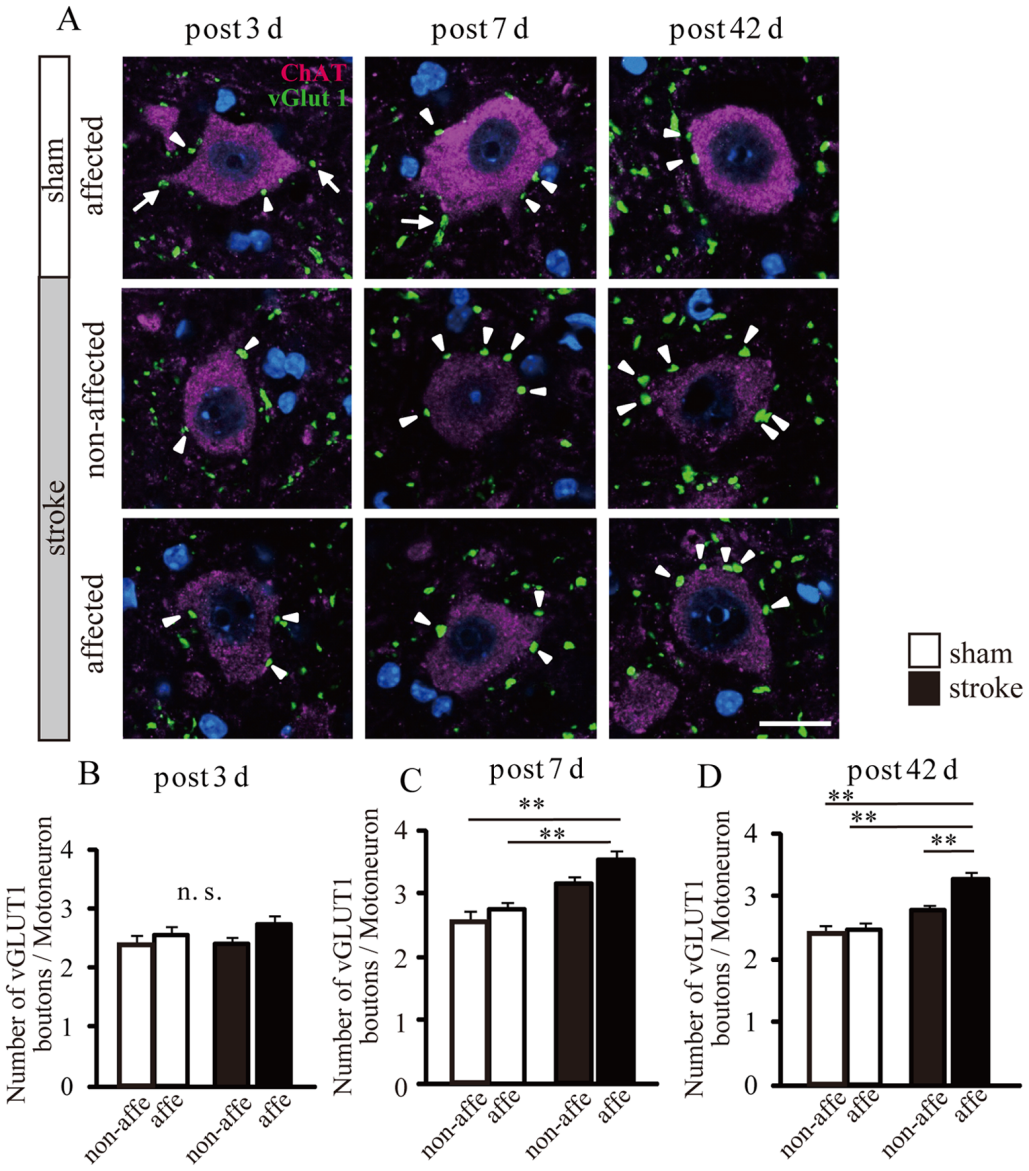
The number of vGluT1-positive boutons on motoneurons after stroke. A: Dual labeling of vGluT1 (green) and ChAT (magenta) at 3, 7, and 42 d after stroke. Arrowheads show vGuT1-positive boutons contacting motoneuron somata and the arrows show non-counted boutons because the boutons did not contact the somata. Scale bar = 20 “Insert>Symbols” µ m. B-D: Quantification of the number of vGluT1-positive boutons on plasma membranes of spinal motoneurons in sham and stroke mice at 3, 7, and 42 d after stroke. Error bars on graphs represent S.E.M. One-way ANOVA with post hoc Tukey-Kramer test, **p<0.01.

ChAT-positive areas were largely found in the cytosol of motoneurons, and we counted the number of vGluT1-positive boutons that merged with ChAT-labeled motoneuronal soma (50 motoneurons in affected and non-affected sides/animal, Fig. 6A-D). The number of vGluT1-positive boutons in the affected side of stroke mice was significantly increased compared to the affected and non-affected sides of sham animals at 7 and 42 d post-stroke (7 d: sham-affected: 2.7±0.1, sham non-affected: 2.6±0.1, stroke-affected: 3.5±0.1, stroke non-affected: 3.1±0.1, 42 d: sham-affected: 2.4±0.09, sham non-affected: 2.3±0.1%, stroke-affected: 3.2±0.1, stroke non-affected: 2.7±0.1%, p<0.01, Fig. 6C, D); however, there was no difference in the affected and non-affected sides between sham and stroke mice at 3 d post-stroke (3 d: sham-affected: 2.5±0.1, sham non-affected: 2.4±0.1, stroke-affected: 2.7±0.1, stroke non-affected: 2.4±0.1, Fig. 6B). In addition, at 42 d post-stroke, the number of vGluT1-positive boutons in the stroke-affected side was significantly increased compared with the non-affected side (p<0.01, Fig. 6D).

## Discussion

The present study revealed decreased KCC2 expression and S940 phosphorylation in KCC2 in the plasma membrane of motoneurons and an increased number of vGluT1-boutons on spinal cord motoneurons following stroke in the rostral and caudal forelimb motor area. This study is the first attempt to determine the mechanisms that underlie post-stroke spasticity in mice.

Spasticity is characterized by a hyper-excitable stretch reflex and increased muscle tone. It has been reported that spasticity in patients with stroke indicates decreased RDD of the H reflex [33]–[34]. Therefore, in the current study, we confirmed spasticity after stroke by electrophysiologically assessing the RDDs of H reflexes (Fig. 2). The RDD of the H reflex is considered to be caused by presynaptic and motoneuron excitability. It is known that repetitive firing of synapses leads to a temporary decrease in synapse strength [35], possibly due to a decrease in presynaptic Ca^2+^ current [36], vesicle depletion [35], postsynaptic receptor desensitization [37]–[38], activity-dependent decreases in neurotransmitter release probability [39]–[40], and action potential conduction failure in the postsynaptic neuron. Our results demonstrated that spasticity was already present 3 d post-stroke and continued until 42 d post-stroke (Fig. 3). This shows that post-stroke, spinal motoneurons exhibited increased excitability even in the acute stage. Previous physiological studies have reported that one of the mechanisms of hyperreflexia in patients with stroke is increased motoneuron excitability. It is known that plateau potentials in motoneurons induced by persistent inward currents can drastically change their intrinsic excitability, and that persistent inward currents are reportedly enhanced in the upper limbs of patients with spastic stroke [41]. However, Mottram et al. demonstrated that persistent inward currents-induced plateau potentials were not observed in spastic-paretic motoneurons; rather, they were due to low levels of spontaneous firing in motoneurons caused by synaptic input to the resting spastic-paretic motoneuron pool [42]–[43]. Although other factors, such as the serotonin receptor 5-HT2C, can cause motoneuron hyperexcitability after spinal cord injury [44], we hypothesized that one cause of motoneuron excitability was a down-regulation of KCC2 in the motoneuron plasma membrane.

KCC2 is located in the plasma membrane of cell somatas, dendritic shafts, and spines in various neuron subtypes [31]. KCC2 functions as a major chloride extruder, which allows GABA_A_ and glycine receptors to function. Moreover, a recent study demonstrated a morphogenic role of KCC2 in spine formation, independent of its ion transport function [45]. However, the role of KCC2 in the dendritic shaft has not been clarified [31]. KCC2 molecules demonstrate monomeric and oligomeric organization with molecular masses of ∼130 to 140 kDa and>200 kDa bands, respectively [27], [46]. KCC2 mRNA translation is not a major rate-limiting step in the regulation of KCC2 expression [47]–[48]. A previous study reported that spinal cord injury-induced down-regulation of KCC2 in motoneurons led to spasticity [17]. In the present study, the decrease of KCC2 expression in the plasma membrane of motoneurons on the affected side was shown early and was also shown to be temporary by immunohistochemical (Fig. 4) and western blot studies (Fig. 5). This is because KCC2 expression on the stroke-affected side was found to be recovered to normal levels by 21 and 42 d post-stroke. On the other hand, a strong down-regulation of KCC2 has also been detected at 7 d after spinal cord injury, and the decline continued until at least 45 d after injury [17]. We also determined that oligomeric KCC2 in the plasma membrane of the stroke-affected side was significantly dephosphorylated at 3 and 7 d post-stroke by western blot (Fig. 5E, H). A previous study demonstrated that PKC-mediated regulation of S940 phosphorylation in KCC2 may be involved in spasticity in the mouse model of spinal cord injury [15]–[16]. Therefore, it is possible that motoneurons affected by stroke show elevated excitability in the acute phase of stroke because the decrease in KCC2 function alters the actions of GABA and glycine. Although KCC2 positive areas were significantly reduced in stroke affected side at 3 d post-stroke and stroke non-affected side at 7 d post-stroke compared to sham animals in immunohistochemical analysis, however, similar results were not detected in western blot analysis (Figs. 4 and 5). This difference between results may have been caused by samples being collected from the ventral horn of the spinal cord for western blot analysis. In other words, we might have extracted solutions containing membrane-enriched fractions of both cell membranes, and also dendrite shafts. As we can specifically analyze the KCC2-positive area in the cell membrane by immunohistochemical analysis, we determined that this approach was more sensitive than western blot analysis.

KCC2 down-regulation was not detected in the affected side at 21 and 42 d post-stroke in western blot and immunohistochemistry studies (Figs. 5 and 4), even though H reflex RDDs were significantly decreased in the affected side at the same time point (Fig. 3C). Our previous study examined the excitability of affected motoneurons with c-Fos immunostaining until 28 d post-stroke. However, at 56 d after stroke, we found that excitability was similar to that of control animals [12]. Thus, we hypothesized that primary afferent fiber sprouting in spinal circuits were over-connected in motoneurons in the chronic stroke phase [8].

Ia afferent fibers, which have muscle spindle primary endings, monosynaptically project to homonymous motoneurons. These fibers are also differently sensitive to presynaptic inhibition. Monosynaptic pathways facilitate the H reflex, and animals with pyramidal tract injury exhibit hyperreflexia [8], although there is no report of this occurring after stroke. Presynaptic Ia inhibition is known as one of inhibition pathways of the H reflex, and this reduction causes hyperreflexia in patients with spinal cord injury. However, Faist et al. demonstrated that paraplegics with unilateral cerebral injury do not exhibit reduced presynaptic Ia inhibition in soleus muscles [49]. Lamy et al. also reported that although the impairment of presynaptic Ia inhibition in patients with stroke behaved similarly in the upper and lower limbs, reduced presynaptic Ia inhibition was more marked at cervical rather than at lumber segments [33]. In the current study, we investigated the number of vGluT1-positive boutons in monosynaptic connections with motoneurons and observed an increased number of projections from Ia afferent fibers after stroke (Fig. 6). VGluT1-positive fibers in the spinal cord are thought to belong mainly to corticospinal and reticulospinal tracts, and Ia, II, and Ib fibers [50]–[52]. These various tracts and fibers project to different areas in Rexed laminae. VGluT1-positive corticospinal and reticulospinal tracts project to the dorsal horn and laminae VII of the medial ventral horn, respectively [51]. Other myelinated vGluT1-positive fibers that project to laminae III-VI are thought to be cutaneous myelinated afferents [53]. Furthermore, Ia afferent fibers project to laminae VII and IX and connect to motoneurons [54]–[55]. Thus, previous studies investigated the number of vGluT1-positive boutons connecting to motoneurons as a way to count Ia afferent fibers [8]. We found that vGluT1-positive boutons of the affected side were significantly increased 7 and 42 d post-stroke compared to sham-operated animals (Fig. 6C, D). Moreover, these increased Ia afferent boutons were excitatory synapses, suggesting that the input from Ia fibers to motoneurons was amplified. We suggest that this increase in Ia boutons is a chronic change, characteristic of spasticity at the cellular level. Furthermore, we suggest that this may be a maladaptive form of plasticity that leads to development of spasticity after stroke.

In this study, transient KCC2 downregulation and dephosphorylation of S940 in KCC2 was detected in the early phase post-stroke. We also observed an increase in the number of vGluT1 boutons until 42 d post-stroke. We speculate that KCC2 expression changes may serve as a trigger of spasticity after stroke, and that other mechanisms of spasticity may exist in stroke. If the increased Ia boutons that connect to motoneurons are also functional, then it might be expected that the spinal reflex would be hyper-excitable. Therefore, axon sprouting and an increase of Ia boutons could cause chronic spasticity after stroke.

The results of the present study suggest that in the motor area post-stroke, there seems to be a decrease in KCC2 expression in the plasma membrane of motoneurons and increased projections of Ia afferent fibers to motoneurons. Moreover, this increase in Ia fibers may be responsible for the expression of chronic phase spasticity after stroke. Studies such as these are important since a better understanding of the mechanisms of spasticity could aid in the development of more effective treatments to promote functional recovery after stroke.
